# Case Report: Homozygous Pathogenic Variant P209L in the *TTC21B* Gene: A Rare Cause of End Stage Renal Disease and Biliary Cirrhosis Requiring Combined Liver-Kidney Transplantation. A Case Report and Literature Review

**DOI:** 10.3389/fmed.2021.795216

**Published:** 2021-12-10

**Authors:** Giuseppe Gambino, Concetta Catalano, Martina Marangoni, Caroline Geers, Alain Le Moine, Nathalie Boon, Guillaume Smits, Lidia Ghisdal

**Affiliations:** ^1^Department of Nephrology, Dialysis and Renal Transplantation, Erasmus Erasme Hospital, Free University of Brussels, Brussels, Belgium; ^2^Department of Genetics, Erasme Hospital, Free University of Brussels, Brussels, Belgium; ^3^Department of Nephropathology, Universitair Ziekenhuis Brussel, Brussels, Belgium; ^4^Department of Nephropathology, Brugmann University Hospital, Brussels, Belgium; ^5^Department of Nephrology, Dialysis and Renal Transplantation, Erasme Hospital, Free University of Brussels, Brussels, Belgium; ^6^Department of Gastroenterology, Erasme Hospital, Free University of Brussels, Brussels, Belgium; ^7^Department of Genetics, Erasme Hospital, Free University of Brussels, Brussels, Belgium; ^8^Department of Nephrology and Dialysis, Epicura Hospital, Saint-Ghislain, Belgium

**Keywords:** ciliopathies, *TTC21B*, clinical exome, end stage renal disease (ESRD), biliary cirrhosis, combined liver and kidney transplant

## Abstract

**Background:** Ciliopathies are rare diseases causing renal and extrarenal manifestations. Here, we report the case of a ciliopathy induced by a homozygous pathogenic variant in the *TTC21B* gene.

**Case Description:** A 47-year-old patient started hemodialysis for chronic kidney disease (CKD) of unknown origin. She presented with early onset of hypertension, pre-eclampsia, myopia and cirrhosis. Renal biopsy showed mild interstitial fibrosis, tubular atrophy, and moderate arteriosclerosis while liver pathology demonstrates grade B biliary cirrhosis. Family history revealed several cases of early-onset severe hypertension and one case of end-stage renal disease (ESRD) needing kidney transplantation at twenty years of age. Clinical exome sequencing showed homozygosis for the pathogenic variant c.626C>T (p.Pro209Leu) in the *TTC21B* gene. The patient underwent combined liver-renal transplantation with an excellent renal and hepatic graft outcome.

**Conclusions:** *TTC21B* gene mutations can lead heterogeneous to clinical manifestations and represent an underappreciated cause of ESRD. The paradigm in diagnosis of CKD of early onset and/or of unknown origin is changing and genetic counseling should be performed in all patients and families that meet those criteria. Renal or combined liver-renal transplantation represents the best option for patients suffering from those diseases in terms of prognosis and quality of life.

## Introduction

Ciliopathies are wide and heterogeneous range of human disorders that are caused by cilia dysfunction. Cilia and their components have a key role in multiple human functions including the perception of environmental cues and the development of many vertebrate tissues. Although the term ciliopathy has been used for the first time in 1984, mechanisms related to cilia dysfunctions are not yet completely understood.

Two orders of ciliopathies have been described according to the localization and function of the muted protein. First order ciliopathies are related to dysfunction of a protein localized or functionally connected with the basal body and/or ciliary compartment. Second order ciliopathies are caused by mutations in proteins that are not part of cilium structure and that indirectly interact with cilium formation or function (1).

The *TTC21B* gene is situated in the short arm of chromosome 2 (2q24.3) and codes for an intraflagellar transport-A (IFT-A) ciliary protein called IFT139. The intraflagellar transport (IFT) complex is a group of at least 20 proteins that were discovered in the 1990s, named according to their molecular weight, and which act as adapters between the motor proteins required for movement and ciliary cargo proteins. IFT complexes (IFT-B and IFT-A) are involved in anterograde and retrograde transport respectively (2) and defects in IFT proteins typically disrupt ciliary assembly and attenuate Hedgehog signaling which is a highly conserved intracellular pathway involved in in embryogenesis and organ development (3). IFT complex mutation leads to first order ciliopathies (2).

**Figure 1:**
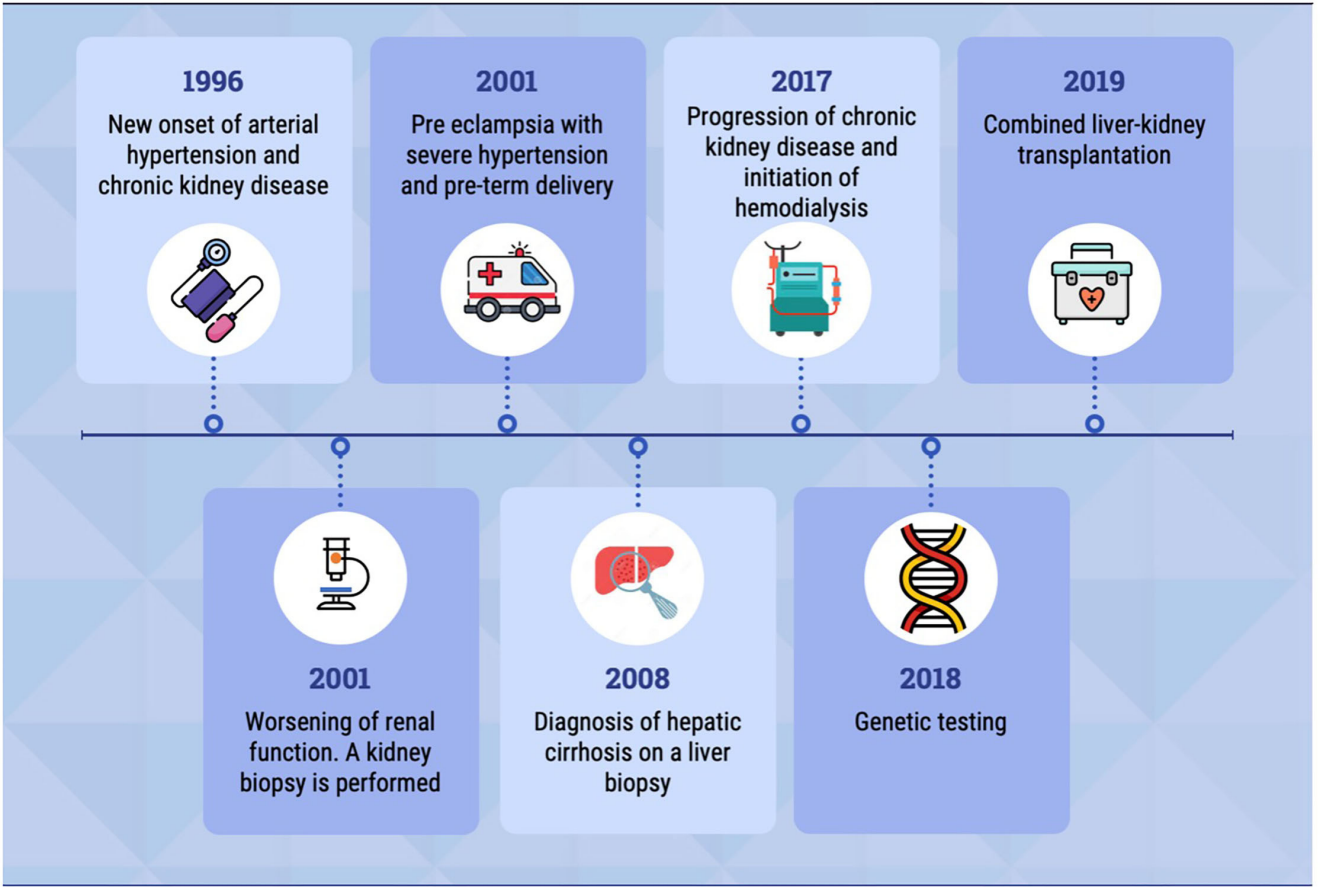
The timeline shows the most relevant events in patient's clinical history.

Here, we report a case of a homozygous pathogenic variant in the *TTC21B* gene, with renal and extrarenal manifestations. The patient's clinical history is summarized in the timeline (Figure 1).

## Case Description

A 47-year-old woman was referred to the nephrology department in February 2017, in order to initiate haemodialysis for end-stage renal disease (ESRD).

**Figure 2:**
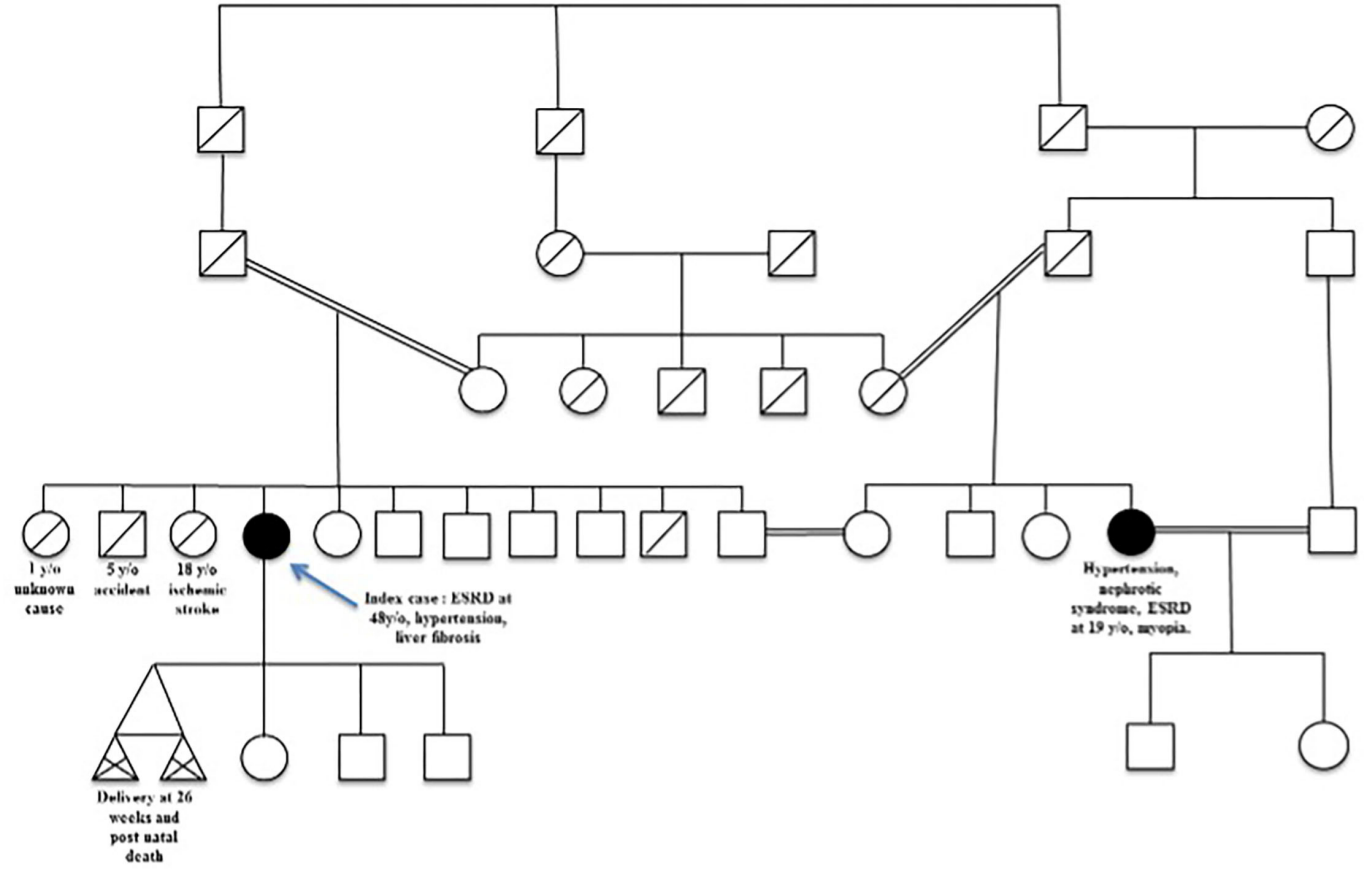
Patient's familial pedigree. The index case is indicated by the blue arrow.

She was diagnosed with severe hypertension and chronic kidney disease (CKD) at age 20. She was born from an inbred union of a North African family and several cases of hypertension, myopia, and severe kidney disease occurred in the extended family. Her past medical history was unremarkable except for obesity. The genealogical tree (Figure 2) showed several loops of consanguinity predisposing to recessive disease. Her brother died at age 5 and one of her sisters died from stroke at age 18 due to severe hypertension. A maternal cousin, also born from an inbred union, presented with myopia, nephrotic syndrome, ESRD, requiring dialysis and renal transplantation at age 20.

At diagnosis, serum creatinine was 1.4 mg/dL. Urinary sediment was within normal limits, while a moderate mixed proteinuria was detected with a protein/creatinine ratio of 1.5 g/g. Auto-immunity screening was negative, but complement was not investigated. Renal ultrasound revealed no abnormalities. Renal magnetic resonance imaging (MRI) ruled out renal arterial stenosis, but no other tests aimed to exclude secondary hypertension were available in the patient's medical record. Angiotensin-converting enzyme inhibitor was started as the only treatment.

**Figure 3:**
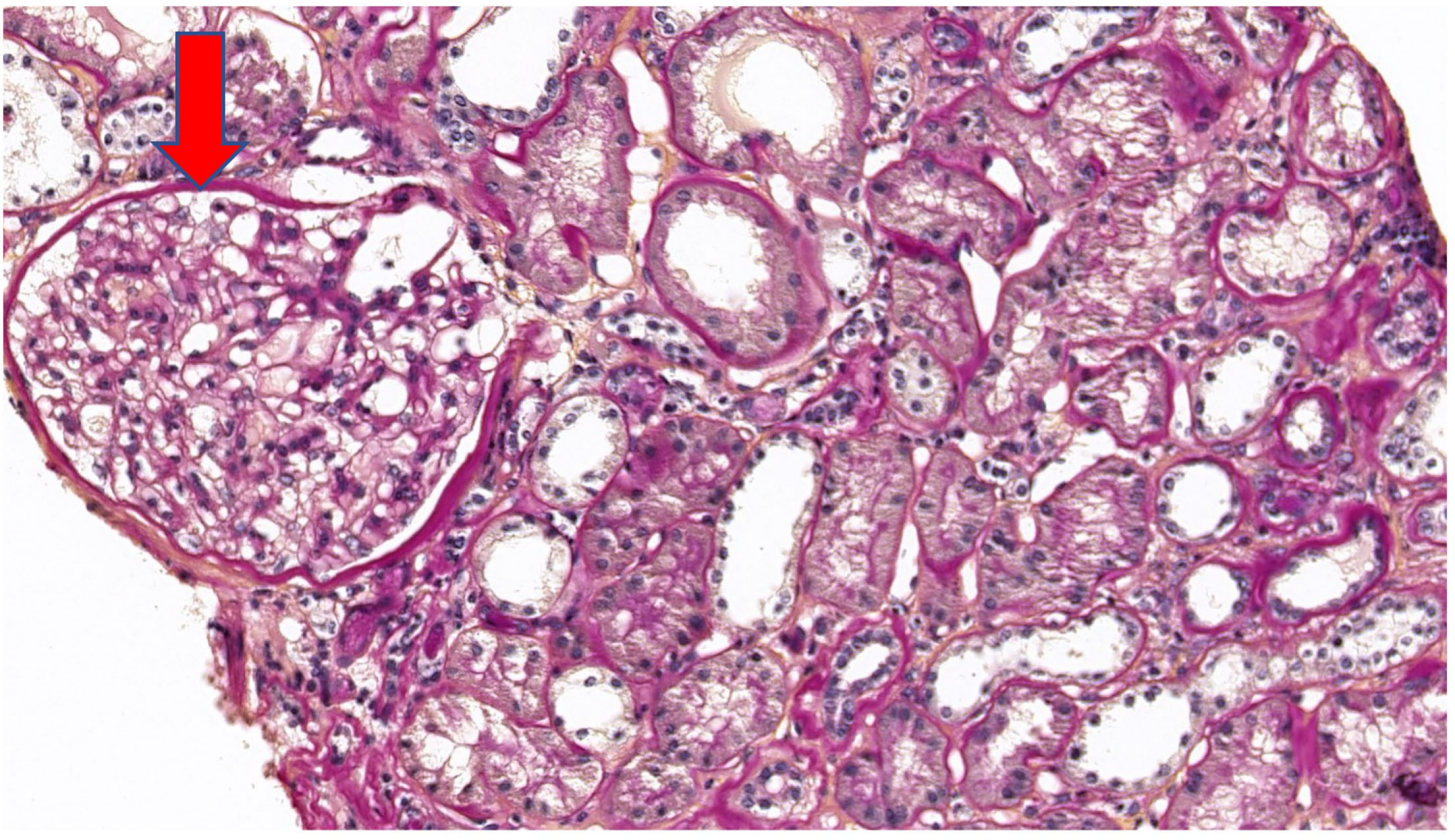
Periodic acid Schiff (PAS) stain at ×20 magnification on light microscopy showing a normal glomerulus (arrow).

**Figure 4:**
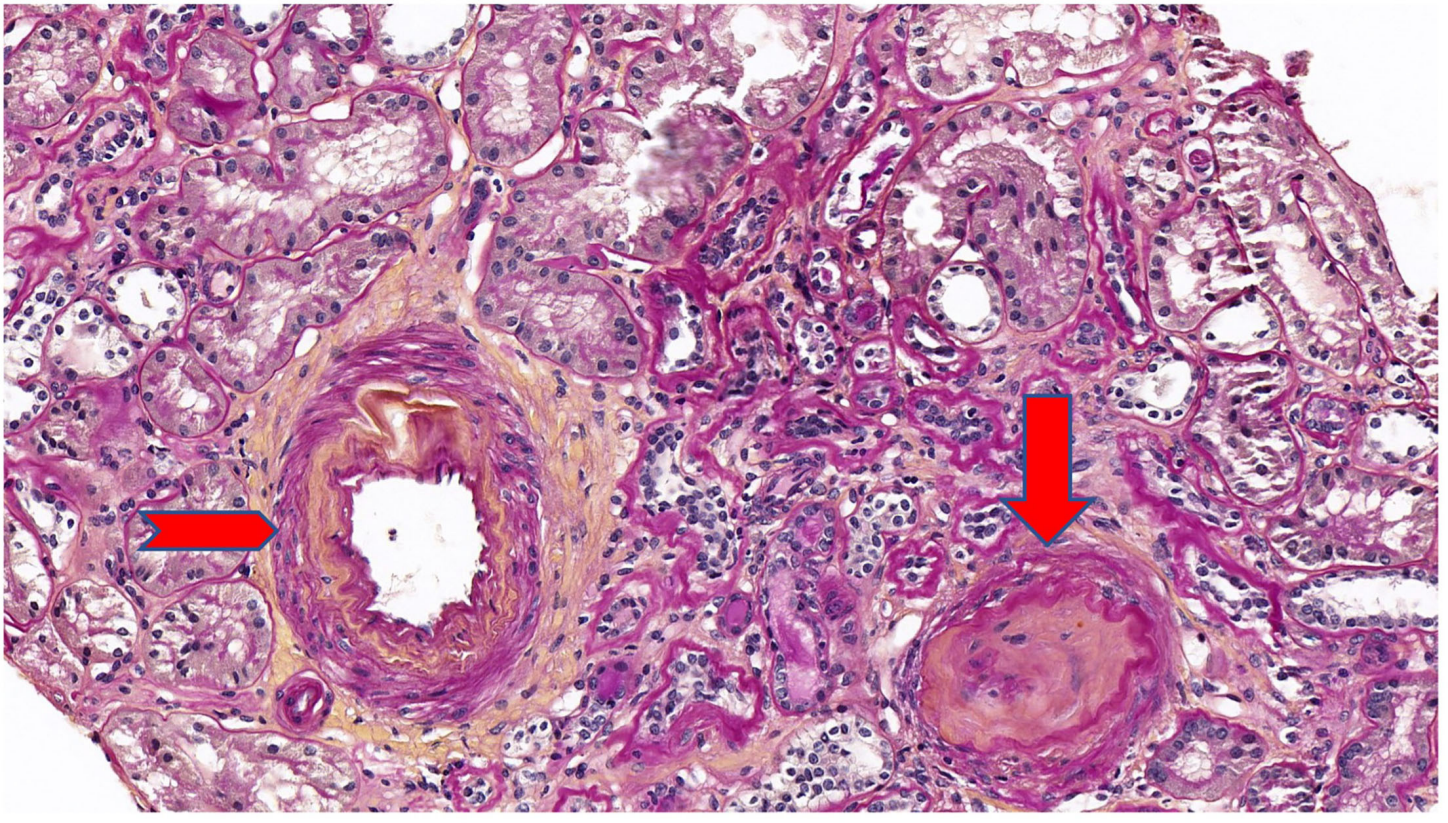
Periodic acid Schiff (PAS) stain at ×20 magnification on light microscopy showing a mild interstitial fibrosis and tubular atrophy. One glomerulus out of 6 showed a complete sclerosis (arrow). Arteries showed a moderate arteriosclerosis (arrowhead).

Five years after diagnosis, a serum creatinine of 2.79 mg/dL justified a kidney biopsy. Light microscopy (Figure 3) showed six glomeruli, five of which were within normal limits while one presented advanced sclerosis. Lesions of mild interstitial fibrosis and tubular atrophy were described. Arteries showed moderate arteriosclerosis (Figure 4). Immunofluorescence staining and electron microscopy have not been performed because frozen and glutaraldehyde fixed tissue were not available. An episode of pre-eclampsia occurred during the same year, leading to delivery at 26 weeks and post-natal death of twins.

Concurrently, an isolated cholestasis appeared. Viral serologies and immunological screening were negative. No alpha-1 anti-trypsin (AAT) deficiency was detected. Magnetic resonance imaging (MRI) showed hepatosplenomegaly and hepatic elastography values were consistent with severe fibrosis (64 kPa). Two consecutives liver biopsies showed micro-vacuolar steatosis and periportal fibrosis. Twelve years after diagnosis, because of increasing in liver enzymes and persistent severe itching, a third liver biopsy was performed. It revealed lesions of chronic cholestasis with focal acute cholangitis and peri-portal fibrosis highly suggestive of grade B biliary cirrhosis according to the Child-Pugh score.

Due to irreversible deterioration of renal function and poorly controlled hypertension, the patient started haemodialysis at 48 years of age.

A genetic work-up was performed during pre-transplant screening due to an early-onset of ESRD and liver fibrosis of unknown origin.

**Figure 5:**
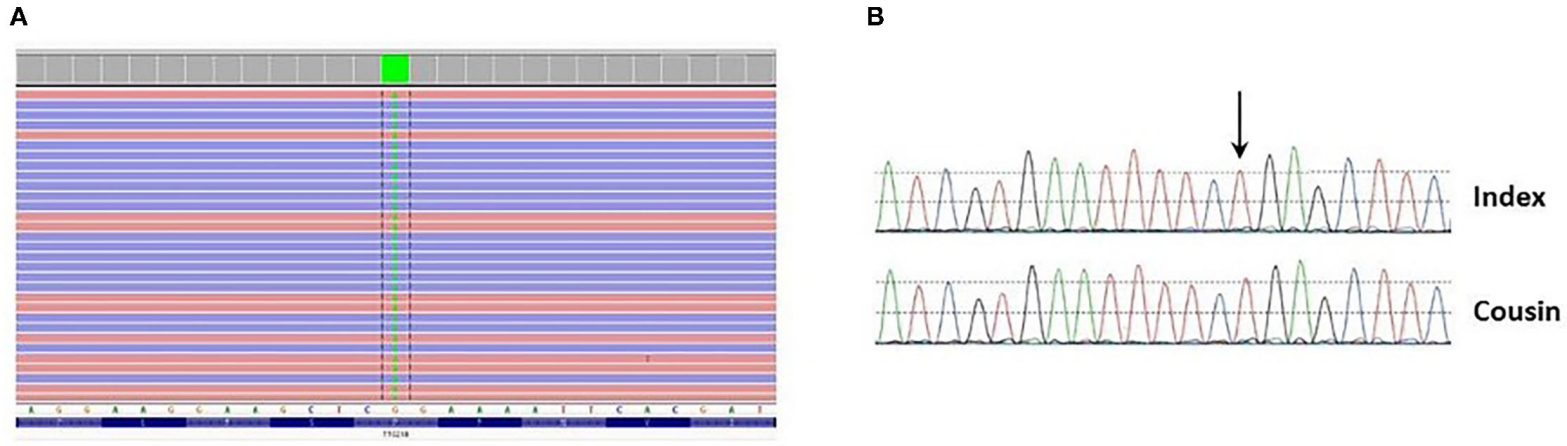
**(A)** Presence of the c.626C>T p.(Pro209Leu) variant (green block) at homozygous state in exon 6 of the TTC21B gene found through clinical exome sequencing and visualized in Integrative Genomics Viewer (IGV). **(B)** Presence of the variant at homozygous state in the patient and her cousin was confirmed by Sanger sequencing.

Clinical exome sequencing was performed on the patient's DNA using in-house SeqCap EZ choice XL capture (Roche Nimblegen, WI, USA) targeting the coding exons of 3989 genes associated with Mendelian disorders. Libraries were sequenced on an Illumina NovaSeq 6000. Variant filtering and interpretation were carried out through Highlander (https://sites.uclouvain.be/highlander/). In particular, 424 genes involved in kidney disease (i.e., cortico-resistant nephrotic syndrome/focal segmental glomerular sclerosis [FSGS], ciliopathies, etc.) were analysed (gene list available upon request). Variant classification was accomplished according to American College of Medical Genetics (ACMG) guidelines (4). Genetic analysis showed that the patient was homozygous for the c.626C>T (p.Pro209Leu) (P209L) variant in the *TTC21B* gene (NM_024753.5) (Figure 5A). The presence of this variant was validated by Sanger sequencing in the patient and her cousin who developed ESRD (Figure 5B). This variant is present with a frequency of 0.012% (34/282510 alleles) and has never been seen in the homozygous state in the gnomAD frequency database (https://gnomad.broadinstitute.org/). Several prediction tools (SIFT, MutationTaster, FATHMM, PolyPhen-2, LRT) agree with the prediction that this change is likely to be deleterious. Moreover, it has been previously reported as pathogenic in several studies (5–8). For these reasons, this variant has been classified as pathogenic (class V).

Combined liver-renal transplantation was performed at 50 of age. Basiliximab has been used as induction therapy, while methylprednisolone, tacrolimus and mofetil mycophenolate have been employed for maintenance. The immediate post-operative period was completely unremarkable. However, few months later the patient developed New Onset Diabetes After Transplantation (NODAT) and pulmonary tuberculosis that recovered without complications after treatment with isoniazid, rifampicin and ethambutol. Both liver and renal graft outcomes remained excellent 24 months after transplantation. The patient experienced substantial improvement in quality of life as she reported repeatedly during transplant follow-up.

At last follow-up, the patient's serum creatinine was 1.4 mg/dL, proteinuria was indetectable (<0.05 g/g creatinine), and urinary sediment was unremarkable. Cytolytic enzymes were within the normal limits (Alanine aminotransferase 20 U/L, Aspartate aminotransferase 26 U/L), as well as albumin (43 g/L) and prothrombin time (10.9 sec; INR 1.04).

Blood pressure was on target without any hypotensive treatment.

## Discussion and Review of the Literature

In the clinical case depicted above, the patient presented with a homozygous missense mutation modifying the amino sequence of the IFT139 protein that is expressed in distal tubules and podocytes.

Animal models in mice and zebrafish have shown that knockdown or missense mutations of this protein can lead to ciliary impairment in tubular renal cells resulting in primary cilia defects, abnormal cell migration, and cytoskeletal alterations (9).

Mutations in *TTC21B* gene lead to a wide spectrum of phenotypes such as syndromic Jeune asphyxiating thoracic dystrophy (JATD) and nephronophthisis (NPHP) with or without extrarenal manifestations. The nephronophthisis phenotype is characterized by reduced renal concentrating ability, chronic tubulointerstitial nephritis, cystic renal disease, and progression to ESRD before the age of 30 (10).

p.P209L homozygous mutations have mostly been described in patients of North African or Portuguese descent indicating a founder effect, and several renal and extrarenal manifestations have been associated to this specific mutation in the currently available literature (Table 1).

**Table 1:** Publications reporting cases with TTC21B p209L mutation.

**References**	**No. of patients**	**Phenotype**	**Renal histology**
Doreille et al. (11)	6	CKD (*n =* 7), history of hypertensive emergency (*n =* 5), hepatic involvement (*n =* 5)	Vascular lesions (*n =* 6), TMA (*n =* 4), no tubule-interstitial lesions or FSGS founded.
Cong et al. (5)	18	CKD (*n =* 18), proteinuria (*n =* 16), hypertension (*n =* 15), hepatic involvement (*n =* 2), cerebral aneurism (*n =* 2), myopia (*n =* 1)	FSGS (*n =* 10), tubulointerstitial fibrosis (*n =* 10)
Bullich et al. (6)	3	CKD (*n =* 2), proteinuria (*n =* 3), hypertension (*n =* 2), myopia (*n =* 2)	Global sclerosis (*n =* 1), FSGS (*n =* 2), Tubulointerstitial fibrosis (*n =* 3)

In particular, Cong et al. have described the association between *TTC21B* p.P209L mutation and hereditary FSGS in ten families (18 cases). Clinical features were late-onset proteinuria, steroid-resistant nephrotic syndrome, FSGS associated with tubulointerstitial lesions, high blood pressure and ESRD occurring between the ages of 15 and 35 (5).

Moreover, Bullich et al. described another three cases with high blood pressure associated to the same homozygous mutation and two other compound heterozygous cases including one p.P209L mutation (3). Out of those 23 cases described in the literature, high blood pressure and myopia were present in 74 and 22% of patients respectively (5, 6).

Doreille et al., have recently reported a series of seven patients with *TTC21B* mutations, six of which presented with a p.P209L homozygous mutation. All those patients presented with severe hypertension (associated with left ventricular hypertrophy and hypertensive retinopathy) and ESRD. Four of them underwent a kidney biopsy showing hypertensive nephrosclerosis with arteriolar thrombotic microangiopathy (TMA), without the usual aspect of nephronophthisis. Two out of seven patients had biologic evidence of TMA.

The authors speculated that hypertension in patients with nephronophthisis could be related to dysfunctional cilia located on endothelium which are key in blood pressure control. They brilliantly coined the term “nephroangionophthisis” to broaden the spectrum of nephronophthisis and to better define the phenotype with vascular injury and hypertension which was nearly unknown before (11).

Therefore, patients affected by *TTC21B* mutations can present with different renal manifestations that may overlap with each other. Phenotypes include not only familial nephrotic syndrome associated with lesions of Focal and Segmental Glomerulosclerosis (FSGS) (10) and tubulo-interstitial abnormalities (2), but also endothelial damage and hypertension with histological and/or biological evidence of TMA (11).

Hepatic impairment is not uncommon in *TTC21B* mutations. Zhang et al., reported one pediatric case with mild cytolysis and one with hepatosplenomegaly, both associated with NPHP. Cong et al., described a case of biliary cirrhosis and one case of chronic chilestasis (5, 12). An association of congenital hepatic fibrosis with NPHP or JATD has also been reported (13). In the series published by Doreille et al. (11), five out of seven patients had liver test abnormalities (mild cholestasis and cytolysis) and MRI showed an aspect of Caroli disease in one patient.

While obesity and diabetes are not typical features of *TTC21B* mutation, a murine model showed that *TTC21B*/*IFT139* knockout mice developed hyperphagic behavior and obesity, resulting in type II diabetes mellitus and fatty liver disease (2).

The renal involvement in our patient was mainly characterized by mild interstitial fibrosis and tubular atrophy. Neither lesions of FSGS nor TMA were seen on kidney biopsy, but the renal sample size was too small to draw conclusions on this point. Extra-renal manifestations included hypertension, obesity, chronic cholestasis and slowly progressive hepatic fibrosis requiring liver transplantation. However, given the degree of inbreeding in the patient's family, the hepatic impairment might be the result of other pathogenic mutations or, less probably, due to non-genetic causes.

The same homozygotic *TTC21B* p.P209L mutation was found after transplantation in the patient's elder cousin who presented with progressive CKD, severe proteinuria and myopia. As one of the patient's sisters died at the age 18 from an ischemic stroke due to severe hypertension, it could be speculated that she was carrying the same mutation, with the nephroangionophthisis phenotype as described by Doreille et al. (11) even if sequencing result for other members of the patient's family, including parents, was not performed. As a result, it cannot be excluded that this pedigree have compound heterozygous mutations containing the c.626C>T and a deletion span of the c.626C>T region that can not be detected by short- read exome and Sanger sequencing.

As treatment is purely supportive, renal or combined liver-renal transplantation represents the best option. It improves survival, especially if liver failure exists, it provides an excellent prognosis because no relapse occurs on the graft, and it ameliorates quality of life due to withdrawal from dialysis. Three cases of combined kidney-liver transplantation (CKLT) have been reported in a French series of pediatric patients suspected of ciliopathy with ESRD and hepatic fibrosis (14). One case of sequential liver-renal transplantation has been described in a juvenile NPHP patient in Japan (15). None of these patients presented with a clear *TTC21B* mutation. To our best knowledge, this is the first case of combined liver-kidney transplantation described in the literature in a genetically proven *TTC21B*-related ciliopathy.

Recent publications underline that the paradigm in diagnosis of CKD of early onset and/or of unknown origin is shifting from precise phenotypic characterization to a focus on genotype. The case described here serves to emphasize the importance of performing genetic analysis in all patients and families that meet testing criteria. Phenotype alone can be misleading for clinicians. In fact, similar disease states with similar pathological findings and can be the expression of unrelated underlying mutations carrying different prognoses. Reporting such cases should be encouraged in order to strengthen the understanding of rare kidney diseases, thereby improving management and patient outcomes.

## Conclusions

*TTC21B* gene mutations like p.P209L are rare but underestimated causes of ESRD. Renal involvement includes NPHP, FSGS and TMA, and can be accompanied by life-threatening extrarenal manifestations such as severe hypertension and/or liver cirrhosis and failure. Renal or combined liver-renal transplantation represents the best treatment option, offering excellent graft outcome and improvement in quality of life. In line with the recent literature (10, 16), we strongly encourage the use of genetic testing and counseling in all cases of chronic kidney disease of early onset and/or of unknown origin.

## Data Availability Statement

The datasets presented in this study can be found in online repositories. The names of the repository/repositories and accession number(s) can be found below: https://www.lovd.nl/, #0000814750.

## Author Contributions

CC contributed to the diagnosis of the disease and to write the manuscript. LG contributed to perform clinical exome sequencing lead to the diagnosis, to write the manuscript, and supervised the whole scientific work. NB and AM contributed to write the manuscript. CG performed the pathology lecture and contributed to write the manuscript. MM and GS contributed to perform clinical exome sequencing and to write the manuscript. GG performed all literature review and contributed to write the manuscript. All authors contributed to the article and approved the submitted version.

## Conflict of Interest

The authors declare that the research was conducted in the absence of any commercial or financial relationships that could be construed as a potential conflict of interest.

## Publisher's Note

All claims expressed in this article are solely those of the authors and do not necessarily represent those of their affiliated organizations, or those of the publisher, the editors and the reviewers. Any product that may be evaluated in this article, or claim that may be made by its manufacturer, is not guaranteed or endorsed by the publisher.
